# Serum metabolomics identifies GPC depletion in hepatic encephalopathy and its therapeutic potential for cognitive impairment

**DOI:** 10.3389/fphar.2026.1807444

**Published:** 2026-04-29

**Authors:** Haoming Chen, Xudan Xing, Xizhi Ding, Guilin Xiang, Jianwei Li, Yi Yang, Qian Liu, Kaiju Xu, Qian Lei, Peng Li

**Affiliations:** 1 Department of Anesthesiology, Chengdu Shang Jin Nan Fu Hospital/Shang Jin Hospital of West China Hospital, Sichuan University, Chengdu, China; 2 Department of Anesthesiology, Sichuan Provincial People’s Hospital, University of Electronic Science and Technology of China, Chengdu, China; 3 School of Medicine, University of Electronic Science and Technology of China, Chengdu, China; 4 Department of Anesthesiology, Wusheng County People’s Hospital, Guangan, China; 5 Anesthesia and Surgery Center, Chengdu Third People’s Hospital, Chengdu, China; 6 General Practice Center, Sichuan Provincial People’s Hospital, University of Electronic Science and Technology of China, Chengdu, China; 7 Department of Anesthesiology, Wenjiang Hospital of Sichuan Provincial People’s Hospital, Chengdu, China; 8 Department of Infectious Disease, Sichuan Provincial People’s Hospital, University of Electronic Science and Technology of China, Chengdu, China

**Keywords:** Alzheimer’s disease, biomarker, cholinergic system, glycerophospholipid metabolism, neuroprotection

## Abstract

**Background:**

Hepatic encephalopathy (HE), a common neurological complication in end-stage cirrhosis, is a complex disorder whose molecular pathogenesis has yet to be fully elucidated. Metabolomics studies provide novel insights into elucidating the pathological mechanisms of HE. This study aims to screen serum differential metabolites in cirrhosis and HE patients using metabolomics technology, with particular focus on validating the therapeutic effects of key metabolites on HE-associated cognitive dysfunction and its underlying mechanisms.

**Methods:**

This study was designed to first enroll 20–30 subjects per group (including healthy controls, cirrhosis patients, and HE patients) for serum untargeted metabolomic analysis using liquid chromatography-tandem mass spectrometry technology, combined with multivariate statistical analysis and pathway enrichment methods to identify key metabolites. This study established five experimental rat groups and developed a HE rat model via modified common bile duct ligation to evaluate the effects of key metabolite supplementation on cognitive function, while simultaneously assessing therapeutic outcomes through histopathological examination and ultrastructural observations.

**Results:**

A total of 2,375 metabolites were identified by metabolomic analysis. Comprehensive analysis screened five differentially expressed metabolites (including sn-glycero-3-phosphocholine (GPC)) significantly associated with disease progression, with GPC prioritized for subsequent validation of its effects on cognitive function in HE. The results demonstrated that GPC supplementation notably improved HE rats’ spatial working memory and motor ability, while alleviating hepatic inflammatory infiltration and blood-brain barrier damage.

**Conclusion:**

This study is the first to discover and confirm the protective effect of GPC in improving cognitive dysfunction in HE, and the mechanism may be related to preserving the integrity of the blood-brain barrier.

## Introduction

Liver cirrhosis is one of the most common global diseases. In the early stages, due to the liver’s compensation, patients may be asymptomatic or exhibit only mild symptoms. Although no obvious clinical manifestations are present, significant metabolic changes may already be occurring within the body (Ginès et al., 2021; Smith et al., 2019). Hepatic encephalopathy (HE) is a neuropsychiatric syndrome caused by acute or chronic liver disease and is a common complication in the late stages of cirrhosis (Weissenborn, 2019; Rudler et al., 2021). The onset of HE significantly increases patient mortality and medical costs, making early and proactive prevention critically important (Rose et al., 2020; Maharshi and Sharma, 2024).

The exact pathogenesis of HE remains incompletely understood. While the traditional ammonia toxicity hypothesis is widely accepted, clinical observations have shown that blood ammonia levels do not always correlate with HE severity, suggesting that other metabolic disturbances may contribute to the disease process (Wang et al., 2023; Jalan and Rose, 2022). As the central metabolic organ of the human body, liver dysfunction can lead to systemic metabolic disorders. In recent years, advances in untargeted metabolomics technology have provided a powerful tool for systematically investigating liver-related diseases (Yu et al., 2024). However, existing research on HE has primarily focused on screening metabolic biomarkers, with insufficient exploration into functional validation of these metabolites and their therapeutic potential (Pelle et al., 2022).

This study aims to elucidate key metabolic changes during the progression from cirrhosis to HE. Furthermore, functional validation of selected metabolites will be conducted, aiming to provide new theoretical insights and therapeutic targets for early metabolic intervention in HE.

## Methods

### Study design and populations

This study planned to enroll healthy control subjects undergoing routine physical examinations, hospitalized patients with compensated liver cirrhosis, and type C HE patients treated at Sichuan Provincial People’s Hospital from January 2024 to August 2024. This study has been approved by the Ethics Committee of the Sichuan Provincial People’s Hospital, with ethical approval number: Ethics Review (Research) No. 43 of 2024. The project has been registered with the Chinese Clinical Trial Registry, registration number: ChiCTR2400089932. All patients participating in the study were informed of the research content and provided signed informed consent. The inclusion criteria of normal control group included: (1) Age between 18 and 80 years; (2) Normal cognitive function as assessed by Mini-Mental State Examination; (3) No underlying chronic diseases (e.g., hypertension, diabetes); (4) No regular medication use; (5) Alcohol consumption ≤50 g/day; (6) Non-pregnant and non-lactating status. The cirrhosis group inclusion required: (1) Age between 18 and 80 years; (2) Confirmed diagnosis of liver cirrhosis (including but not limited to diagnosis by abdominal ultrasound examination and liver biopsy); (3) Compensated cirrhosis status, Child-Pugh class A, with mild esophageal varices allowed but no complications such as ascites or hepatic encephalopathy (HE). Exclusion criteria included: (1) Patients with metabolic-related disorders (including hypertension, diabetes mellitus, and thyroid diseases); (2) History of gastrointestinal bleeding within the past 2 weeks with hemoglobin concentration <90 g/L; (3) Pregnant or lactating women; (4) Individuals with impaired mental status (including psychiatric disorders, lack of self-control ability, or inability to communicate clearly); (5) Patients deemed ineligible for the clinical trial by investigators due to other reasons; (6) Illiterate individuals. HE group participants were adults aged 18–80 years with clinically diagnosed type C HE (confirmed through comprehensive evaluation of medical history, physical examination, laboratory tests, and imaging studies) and absence of pre-existing neurological disorders. Approximately 20–30 samples will be collected for each group (this sample size was determined based on the minimum number of biological replicates required for metabolomics analysis (Billoir et al., 2015)).

### Blood sample collection, processing, and metabolomics analysis

Fasting venous blood samples (5 mL) were collected from participants at 7:00 a.m. daily. Following collection, the blood samples were transferred to centrifuge tubes and allowed to stand at room temperature for 30 min to facilitate clotting and stratification. Subsequently, the samples were centrifuged at 3,500 rpm for 10 min at room temperature. The supernatant was then carefully transferred to clean cryovials and stored at −80 °C. For untargeted metabolomics analysis, standardized sample preparation protocols were employed, followed by liquid chromatography-tandem mass spectrometry (LC-MS/MS) using a Waters ACQUITY UPLC I-Class plus/Thermo QE HF. Chromatographic separation was achieved using an ACQUITY UPLC HSS T3 column (100 mm × 2.1 mm, 1.8 μm) maintained at 45 °C. The mobile phase consisted of (A) water containing 0.1% formic acid and (B) acetonitrile, with a flow rate of 0.35 mL/min. The injection volume was 3 μL (detailed mass spectrometry parameters are provided in Supplementary Table S1).

### Animal grouping, modeling methods and interventions

This study used SPF-grade 10-week-old male SD rats (330–350 g) divided into five groups: normal control group, sham operation (Sham) group, HE group, HE + normal saline (NS) group, and HE + GPC group. The type C HE model was established using a modified common bile duct ligation (CBDL) method, with the end of the fifth week set as the modeling observation endpoint (DeMorrow et al., 2021). The Sham group underwent surgical exploration without bile duct ligation, while the normal control group received no interventions (detailed modeling methods are provided in Supplementary Material). GPC supplementation was administered at a dose of 200 mg/kg via intraperitoneal injection once daily until specimen collection (Muccioli et al., 1996; Schettini et al., 1992). The HE + NS group received normal saline in volumes equivalent to the GPC injection via intraperitoneal injection once daily until specimen collection.

### Behavioral assessments and specimen collection

After 1 week of acclimatization in the animal facility, behavioral tests were conducted prior to model establishment. The second behavioral assessment was performed at the end of the fifth week post-modeling, followed by euthanasia for collection of liver and brain tissue specimens (Rats were placed in a transparent induction chamber and anesthetized with 8% sevoflurane at a flow rate of 2 L/min. After the loss of the righting reflex and the establishment of slow, deep respiration, the depth of anesthesia was confirmed by the absence of a pedal withdrawal reflex. Subsequently, the rats were immediately euthanized by cervical dislocation). Spatial working memory (assessed by spontaneous alternation rate) and spatial reference memory (assessed by the percentage of entries into the novel arm) were evaluated using the Y-maze test, while locomotor activity (assessed by total movement distance) and anxiety-like behavior (assessed by duration spent in the center area) were evaluated using the open field tests (Detailed experimental procedures for the Y-maze and open field tests and criteria for identifying aberrant behavioral data are provided in Supplementary Material).

### Histological analysis

**FIGURE 1:**
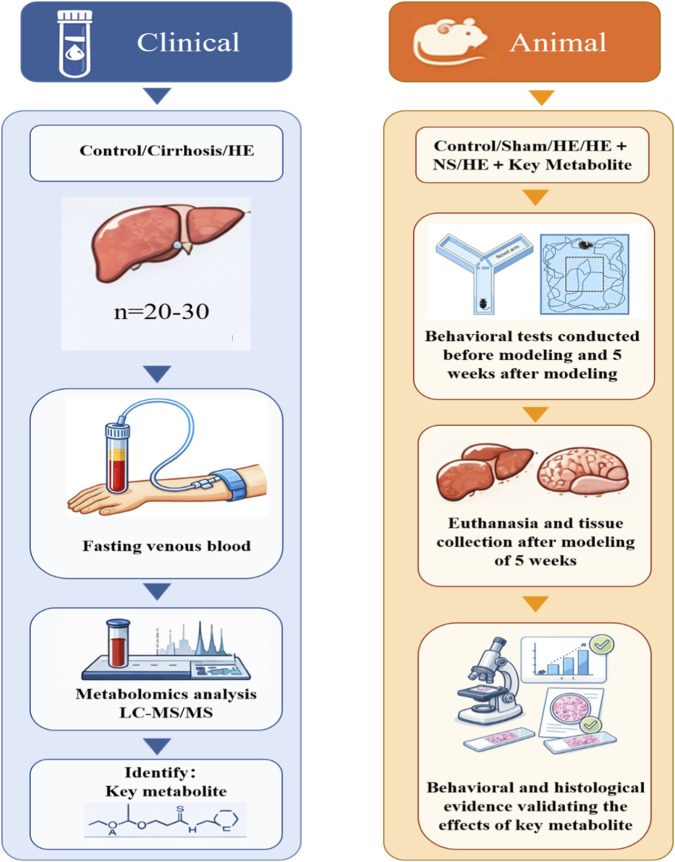
The overall schematic diagram of the experiment.

Liver and brain tissues from rats in each group were subjected to HE staining for histological evaluation. Liver HE staining was performed to verify successful model establishment, while brain HE staining was used to assess the extent of brain injury. Subsequently, the Ishak scoring system was employed to quantitatively assess both the degree of liver fibrosis and the severity of hepatic inflammation (Chowdhury and Mehta, 2023). Transmission electron microscopy was employed to examine the ultrastructure of the blood-brain barrier (BBB) and determine the degree of BBB damage. The entire experimental procedure is shown in Figure 1.

### Statistical methods

Data were analyzed using SPSS 27 software with appropriate statistical methods. Metabolomics data were processed by Progenesis QI v3.0 (Nonlinear Dynamics, Newcastle, UK). Principal component analysis (PCA) and Orthogonal partial least squares-discriminant analysis (OPLS-DA) were used to assess the overall sample distribution and clustering trends, as well as to screen for differential metabolites (Variable importance in projection, VIP >1 and P < 0.05). Pathway enrichment analysis was performed using the KEGG database, while time-series analysis of metabolites was conducted using the Mfuzz R package based on fuzzy clustering principles. Behavioral data were analyzed using Smart 3.0 software (Panlab Harvard Apparatus®), and all graphs were generated using GraphPad Prism 9.0. A P -value <0.05 was considered statistically significant.

## Results

### Significant intergroup differences in laboratory parameters

This study enrolled a total of 83 participants, comprising 30 healthy controls (Con), 30 cirrhosis patients (Cir), and 23 HE patients. Significant intergroup differences (P < 0.05) were observed among all three groups in laboratory parameters including platelet count, prothrombin time, albumin, aspartate aminotransferase, cholinesterase, and bilirubin levels, with progressive deterioration trends correlating with disease progression (Table 1).

**TABLE 1:** Comparison of general information and test results.

Indicators	Con (N = 30)	Cir (N = 30)	HE (N = 23)	P-value
Gender	​	​	​	0.705
Male	17	14	11	​
Female	13	16	12	​
Age (years)	52.067 ± 13.248	55.733 ± 10.586	54.217 ± 12.011	0.496
Hemoglobin (g/L)	137 ± 16.411^**#**^	127.3 ± 23.96 ^**^**^	90.261 ± 23.549	**<0.001**
Platelet count (10^9^/L)	200.5 (96)*^#^	111.5 (77.25) ^**^**^	54 (63)	**<0.001**
PT (sec)	11 (0.88)*^#^	12.75 (1.52) ^**^**^	18.3 (7.9)	**<0.001**
APTT (sec)	27.05 (1.65)^**#**^	28.35 (4.15) ^**^**^	37 (13.3)	**<0.001**
Creatinine (umol/L)	64.9 (14.77)	73.1 (19.77)	66 (31.2)	**0.038**
Total protein (g/L)	68.947 ± 5.324^**#**^	65.647 ± 5.485^**^**^	61.117 ± 10.505	**<0.001**
Albumin (g/L)	42.793 ± 4.015*^#^	38.877 ± 4.228^**^**^	29.7 ± 3.299	**<0.001**
AST (U/L)	20 (6.25)*^#^	35.5 (24.25) ^**^**^	63 (63)	**<0.001**
ALT (U/L)	16 (14)*^#^	29.5 (31.25)	40 (60)	**0.001**
LDH (U/L)	181 (46.5)*^#^	189 (71)	259 (92)	**<0.001**
ALP (U/L)	90 (41.5)^**#**^	89.5 (67.5)	115 (71)	**0.044**
GGT (U/L)	21.5 (27.5)*^#^	52 (63.5)	48 (67)	**<0.001**
Cholinesterase (KU/L)	7.13 (1.75)*^#^	6.05 (2.68) ^**^**^	2.96 (2.22)	**<0.001**
Total bile acids (umol/L)	6.4 (6.73)^**#**^	8.9 (13.2) ^**^**^	145.2 (166.4)	**<0.001**
Total bilirubin (umol/L)	10.2 (6.62)*^#^	13.65 (6.4) ^**^**^	161.7 (226.1)	**<0.001**
Direct bilirubin (umol/L)	3.55 (1.78)*^#^	5.75 (4.6) ^**^**^	97.3 (160.8)	**<0.001**

Bold font indicates statistically significant differences.

Abbreviations: PT, prothrombin time; APTT, activated partial thromboplastin time; AST, aspartate aminotransferase; ALT, alanine aminotransferase; LDH, lactate dehydrogenase; ALP, alkaline phosphatase; GGT, gamma-glutamyl transferase. *P < 0.05 for Con group vs. Cir group; #P < 0.05 for Con group vs. HE, group; ^ P < 0.05 for Cir group vs. HE, group.

### PCA and OPLS-DA model effectively discriminates groups and identifies candidate metabolites

**FIGURE 2:**
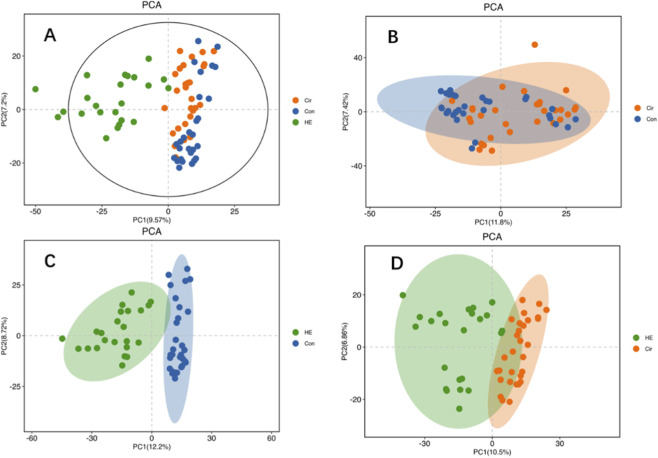
PCA figure. **(A)** All samples - Samples in the Con and Cir groups showed good clustering, indicating low intra-group variability. In contrast, samples in the HE group exhibited poor clustering, suggesting greater inter-individual differences within this group. **(B)** Cir VS Con - Good intra-group aggregation and small inter-group differences. **(C)** HE VS Con - Good intra-group aggregation and large inter-group differences. **(D)** HE VS Cir - Good intra-group aggregation and relatively large inter-group differences.

**FIGURE 3:**
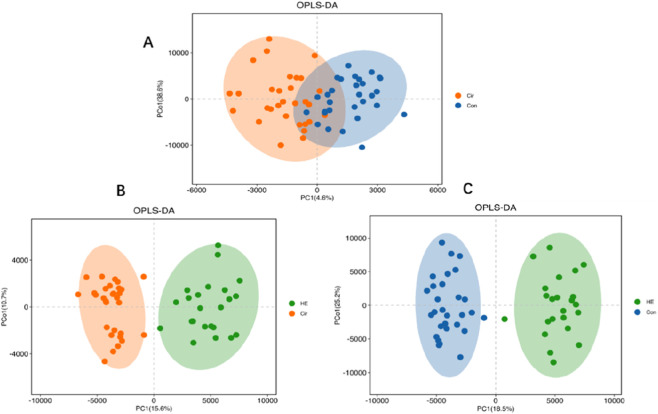
OPLS-DA figure. **(A)** Cir VS Con - partial intergroup overlap; **(B)** HE VS Cir - good intergroup separation; **(C)** HE VS Con - excellent intergroup discrimination.

**FIGURE 4:**
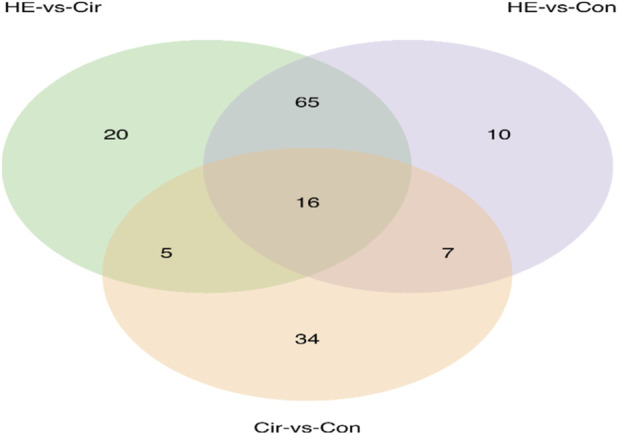
Venn figure. The three comparison groups shared 16 significantly differential metabolites (P < 0.05, VIP >1), among which 10 were Level 1/2 metabolites.

A total of 2,375 metabolites were detected by LC-MS/MS, including 1,053 Level one and Level 2 metabolites. The PCA plot of all samples is shown in Figure 2A. The PCA and OPLS-DA analysis revealed partial overlap between the Con and Cir group (Figures 2B, 3A), while demonstrating good separation between the Cir and HE groups (Figures 2D, 3B). Notably, excellent discrimination was observed between the Con and HE groups (Figures 2C, 3C). Permutation testing confirmed the OPLS-DA model reliability without overfitting (Supplementary Figure S1). Venn diagram (Figure 4) analysis of differential metabolites identified 10 Level 1/2 metabolites. The two metabolic pathways with the smallest *p*-values were glycerophospholipid metabolism and choline metabolism in cancer (Supplementary Figure S2). Time-series analysis revealed that metabolites in Clusters 3, 7, and 10 exhibited the anticipated dynamic changes during disease progression (Supplementary Figure S3). Integrated analysis suggested five metabolites - GPC, phosphatidylcholine(22:6) (PC(22:6)), PC(22:5), PC(17:0), and PC(20:5) - may exert protective effects on cognitive function in liver disease patients (analytical rationale detailed in Supplementary Material).

### GPC improves working memory and motor function in HE rats

**FIGURE 5:**
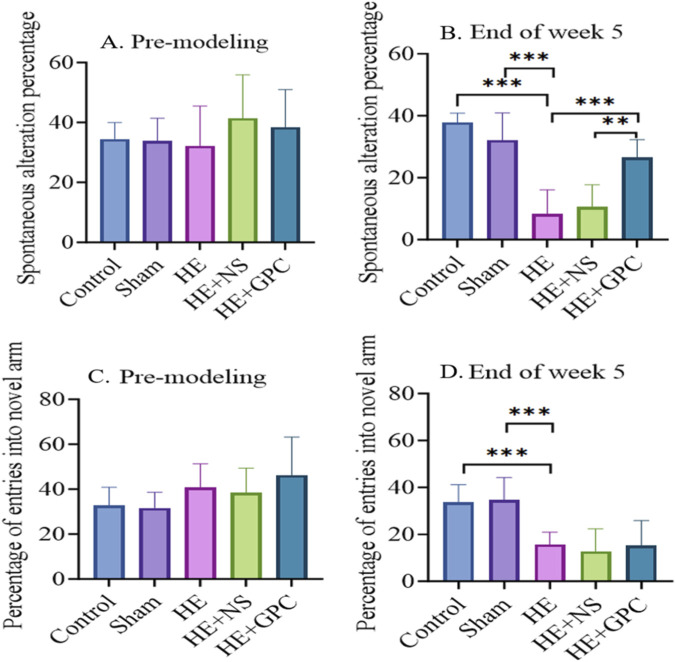
The results of Y-maze test. **(A)** No significant differences were observed in the spontaneous alternation rate among all groups before modeling. **(B)** At the end of the fifth week post-modeling, the HE group exhibited a significantly lower spontaneous alternation rate (8.31% ± 7.75%) compared to both the Control group (37.87% ± 2.94%, P < 0.001) and the Sham group (32.11% ± 8.742%, P < 0.001). In contrast, the HE + GPC group showed a significantly higher spontaneous alternation rate (26.55% ± 5.71%) than both the HE group (P < 0.001) and the HE + NS group (10.48% ± 7.142%, P < 0.01). **(C)** No significant intergroup differences were found in the percentage of novel arm entries before modeling. **(D)** At the end of the fifth week post-modeling, the percentage of novel arm entries in the HE group (15.71% ± 5.204%) was significantly lower than that in the Control group (33.70% ± 7.465%, P < 0.001) and the Sham group (15.15% ± 10.71%, P < 0.001). **P < 0.01, ***P < 0.001.

**FIGURE 6:**
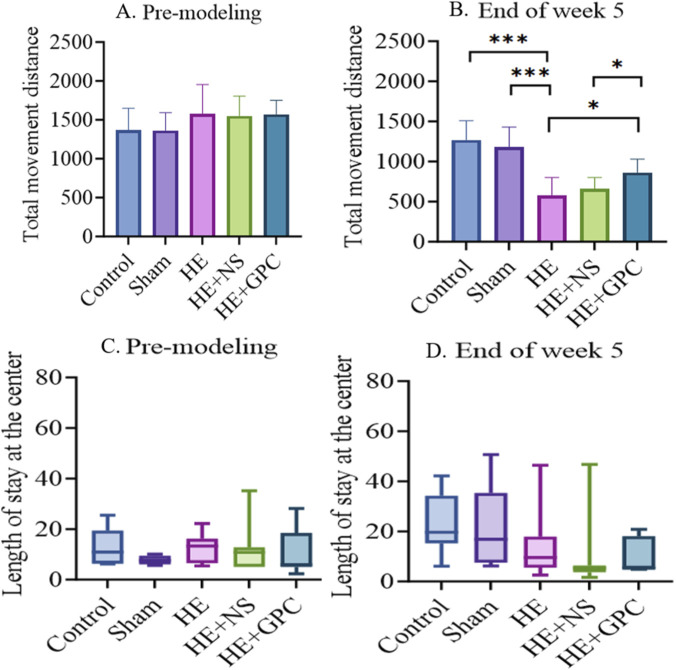
The results of open field test. **(A)** No significant differences were observed in the total movement distance among all groups before modeling. **(B)** At the end of the fifth week post-modeling, the HE group exhibited a significantly shorter total movement distance (573.6 ± 224.4 cm) compared to both the Control group (1,270 ± 238.5 cm, P < 0.001) and the Sham group (1180 ± 250.9 cm, P < 0.001). In contrast, the HE + GPC group showed a significantly longer total movement distance (862.9 ± 167.7 cm) than both the HE group (P < 0.05) and the HE + NS group (656.9 ± 144.7 cm, P < 0.05). **(C)** No significant intergroup differences were found in the length of stay at the center before modeling. **(D)** At the end of the fifth week post-modeling, no significant differences were observed in the length of stay at the center among all groups. *P < 0.05, ***P < 0.001.

During data analysis, the following rat behavioral data were identified as outliers and subsequently excluded from final statistical processing: (1) Rats with unsuccessful model induction at the 5-week endpoint, including those showing no observable jaundice (conjunctival or fur discoloration) and no cirrhotic changes in liver histopathology by HE staining; (2) Rats demonstrating inherently poor baseline cognitive performance during pre-modeling behavioral tests, specifically defined as having either one parameter (spontaneous alternation rate, total movement distance, or cumulative activity) fall below 50% of the group mean while the remaining two parameters are both below 80% of the mean. The Y-maze test at week five showed that the HE group exhibited significantly impaired spatial working memory compared to both the normal control group (P < 0.001) and Sham group (P < 0.001). GPC supplementation improved the spatial working memory (P < 0.001, Figures 5A,B). No statistically significant differences were observed in spatial reference memory among the groups (Figures 5C,D). In the open field test, the HE group demonstrated significantly reduced locomotor activity compared to the normal control group (P < 0.001) and Sham group (P < 0.001). GPC treatment increased the total movement distance (P < 0.05, Figures 6A,B), though no significant effects were observed on anxiety-like behavior (Figures 6C,D).

### GPC reduces inflammation and BBB damage but not cirrhosis or neuronal morphology

**FIGURE 7:**
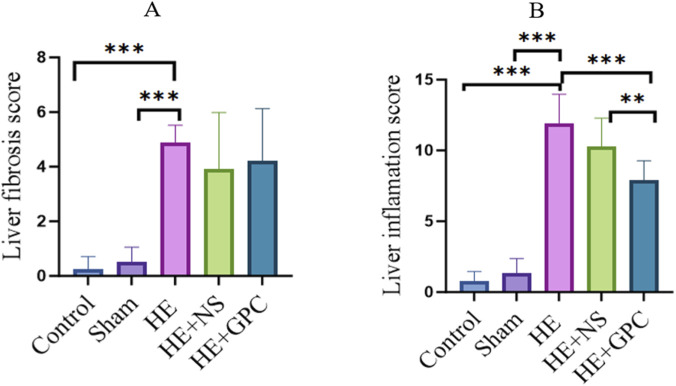
Ishak scores for liver fibrosis and inflammation. **(A)** The liver fibrosis score in the HE group (4.88 ± 0.641) was significantly higher than that in the Control group (0.25 ± 0.463, P < 0.001) and the Sham group (0.5 ± 0.548, P < 0.001). No significant difference was observed in liver fibrosis scores between the HE group and the HE + GPC group (P = 0.36). **(B)** The liver inflammation score in the HE group (11.88 ± 2.1) was significantly higher than that in the Control group (0.75 ± 0.707, P < 0.001) and the Sham group (1.33 ± 1.033, P < 0.001). Notably, the liver inflammation score in the HE + GPC group (7.9 ± 1.370) was significantly lower than that in the HE group (P < 0.001) and the HE + NS group (10.27 ± 2.005, P < 0.001).

**FIGURE 8:**
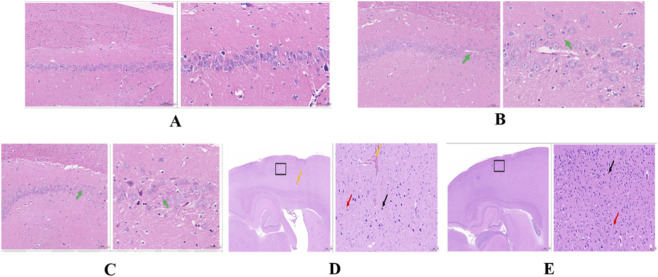
HE results of the brain tissue. Each group comprises two micrographs: for the Control, Sham, and HE groups, the left panel displays ×100 magnification while the right panel shows ×400 magnification, revealing disorganized pyramidal cell arrangement and reduced cell numbers (green arrows); in the HE + NS and HE + GPC groups, the left panel presents a ×20 overview and the right panel shows ×200 magnification (black rectangle indicating the zoomed area), demonstrating multiple shrunken neurons (black arrows) with decreased volume, irregular morphology, indistinct nucleocytoplasmic boundaries, and intensified staining, along with rare necrotic cellular debris (red arrows) and occasional vascular congestion (orange arrows). **(A)** Control. **(B)** Sham. **(C)** HE. **(D)** HE+NS. **(E)** HE+GPC.

**FIGURE 9:**
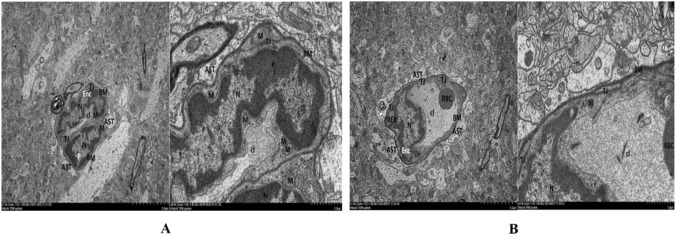
Transmission electron microscopy ultrastructure of the BBB. Compared to the HE + GPC group, the HE + NS group exhibited more severe ultrastructural damage: more indistinct nuclear membranes of endothelial cells; more pronounced mitochondrial swelling with greater dissolution and cavitation of the matrix and cristae; slightly widened intercellular spaces; more obvious atrophy and collapse of the vascular lumen; and nuclear heterochromatin condensation with mitochondrial swelling in pericytes. Enc, endothelial cell; N, nucleus; M, mitochondrion; RER, rough endoplasmic reticulum; TJ, tight junction; P, pericyte; BM, basement membrane; cl, capillary lumen; AST, Astrocytic end-foot; RBC, red blood cell. **(A)** HE+NS. **(B)** HE+GPC.

Liver HE staining images and quantitative analysis revealed significant cirrhosis changes in the HE group compared to Control and the Sham group (Figure 7A; Supplementary Figure S4). Although the degree of cirrhosis showed no marked improvement in the HE + GPC group relative to the HE group (Figure 7A), there was a noticeable reduction in hepatic granulocyte infiltration (Figure 7B; Supplementary Figure S4). Brain tissue HE images demonstrated disorganized pyramidal cell arrangement and decreased cell density in the hippocampal region of HE rats, with GPC intervention failing to significantly ameliorate these neuronal morphological damages (Figure 8). Transmission electron microscopy observations indicated that GPC supplementation significantly alleviated BBB impairment (Figure 9).

## Discussion

The core feature of HE is cognitive dysfunction (Won et al., 2022; López-Franco et al., 2021). Although existing studies have demonstrated the significant role of metabolic disturbances in the pathogenesis of HE, research on key metabolites associated with cognitive function during HE progression and their underlying mechanisms remains limited (Cheon and Song, 2021). In this study, we employed untargeted LC-MS/MS metabolomics technology to systematically analyze serum metabolic changes in Con, Cir, and HE groups, aiming to identify active metabolites related to cognitive function and investigate their potential biological mechanisms.

A total of 2,375 metabolites were detected in this study, including 1,053 Level 1/2 metabolites, demonstrating high metabolite coverage and identification accuracy. The reliability of differential metabolite screening was ensured by employing OPLS-DA models combined with permutation tests, effectively avoiding model overfitting. Differential analysis identified 10 significantly altered metabolites across Con, Cir and HE groups, including five glycerophosphocholine metabolites. These metabolic changes showed strong correlation with disease progression, suggesting their potential roles in cirrhosis and HE, likely reflecting lipid metabolism disorders resulting from impaired liver function (Tadokoro et al., 2023).

KEGG pathway enrichment analysis revealed that these differential metabolites were primarily enriched in glycerophospholipid metabolism and choline metabolism in cancer pathways. The downregulation of glycerophospholipid metabolism may be associated with decreased hepatocyte membrane stability and impaired cellular function, while alterations in choline metabolism in cancer pathways might be linked to neurotransmitter synthesis and cognitive changes (Swapna et al., 2006; Zöllner et al., 2023).

In this study, we ultimately identified five key metabolites for validation, with particular focus on GPC as the most significant candidate. GPC is a naturally occurring water-soluble phospholipid intermediate and choline derivative in humans, composed of choline and glycerophosphate. Serving as a crucial precursor for the synthesis of membrane phospholipids, acetylcholine, and phosphatidylcholines, this low molecular weight compound (257.22 Da) demonstrates excellent intestinal absorption and efficient BBB penetration when administered orally (Wang et al., 2021; Kansakar et al., 2023; Strifler et al., 2016). Although GPC has been approved in several countries as a nutritional supplement or therapeutic agent to improve cognitive, behavioral, and functional outcomes in patients with Alzheimer’s disease, stroke, and cerebral ischemic attacks, its potential effects in liver disease patients remain unexplored in existing literature (Sagaro et al., 2023; Cantone et al., 2024). Notably, significant international variations persist regarding its prescription drug approval status and therapeutic efficacy evaluations. Our study represents the first systematic assessment of GPC’s cognitive protective effects during disease progression from cirrhosis to HE.

The Y-maze test results demonstrated that GPC supplementation significantly improved spatial working memory in HE rats, while showing no protective effect on their impaired spatial reference memory. The preservation of spatial working memory by GPC may be attributed to its short-term enhancement of the cholinergic system in HE rats. GPC potentiates acetylcholine (Ach) synthesis and release, thereby augmenting cholinergic function and consequently improving related behavioral outcomes (Amenta et al., 2006; Giovannini et al., 1998). Notably, while some studies attempting direct choline supplementation for alleviating cognitive symptoms in Alzheimer’s disease showed limited efficacy, GPC has exhibited superior performance in certain investigations. This suggests that GPC’s cognitive-enhancing effects are not mediated solely through its breakdown into choline and subsequent neurotransmitter synthesis (Tayebati and Amenta, 2013). Previous research has further established that GPC upregulates the expression of high-affinity choline uptake transporters, vesicular Ach transporters, and hippocampal choline acetyltransferase, collectively increasing Ach levels (Lee et al., 2017; Jeong et al., 2022). Beyond its effects on cholinergic neurotransmitters, studies have revealed GPC’s influence on cholinergic receptors. Chronic GPC administration in aged rats restored muscarinic M1 receptor densities to juvenile levels (Muccioli et al., 1996). Additionally, GPC may modulate neuronal signaling by affecting membrane functionality, potentially through its role as a phosphatidylcholine precursor that enhances membrane fluidity (Schettini et al., 1992). Covariance analysis confirmed that locomotor activity changes did not significantly influence spontaneous alternation rates. While GPC supplementation partially restored spatial working memory in HE rats, its inability to improve spatial reference memory might reflect the longer-term information storage requirements associated with this cognitive domain.

The results of this study demonstrate that GPC could partially restore locomotor activity in HE rats, while showing no significant effect on their anxiety-like behaviors. These findings are consistent with previous reports regarding GPC’s beneficial effects on motor performance, supporting its potential as a novel sports supplement (Bellar et al., 2015; Marcus et al., 2017). The improvement in motor function may be attributed to GPC’s ability to markedly enhance receptor-stimulated phosphatidylinositol hydrolysis in cortical synaptoneurosomes and significantly increase calcium fluctuations in hippocampal synaptosomes, thereby promoting motor capability (Schettini et al., 1992).

HE images of liver tissue revealed that rats receiving long-term GPC supplementation exhibited reduced hepatic granulocyte infiltration. Previous studies have demonstrated GPC’s ability to downregulate certain inflammatory factors, such as IL-1β expression in the hippocampus (Jeong et al., 2022; Umino et al., 2023), suggesting that the observed reduction in granulocyte infiltration may be attributed to GPC’s anti-inflammatory properties, though this requires further validation. Brain HE images showed that GPC failed to provide direct protection against neuronal morphological damage or brain tissue injury. However, transmission electron microscopy demonstrated that GPC effectively preserved BBB integrity and mitigated BBB disruption—an effect consistent with previous reports (Matsubara et al., 2018). While prior studies confirmed GPC’s ability to reduce both neuronal death and BBB damage in epileptic rats (Lee et al., 2017), the current study only observed BBB protection in HE rats without significant neuroprotection. This discrepancy may stem from the more severe pathophysiological alterations induced by the CBDL model of HE compared to acute seizure models, potentially limiting GPC’s protective efficacy. Although the CBDL model is internationally recognized for modeling type C HE, its profound pathophysiological impact may have obscured GPC’s neuroprotective potential. Future studies employing alternative models, such as quaternary ammonium salt feeding, might better elucidate GPC’s possible neuronal protective effects.

Although this study has revealed metabolic changes and their potential relevance during the progression of cirrhosis and HE, several limitations should be acknowledged. First, the relatively small sample size in the metabolomics analysis per group may have limited the detection of subtle metabolic alterations. Future studies with larger cohorts are needed to validate the reliability of these findings. Second, this study focused solely on serum metabolomic changes. Integrating tissue- or cell-based metabolomics data could provide a more comprehensive understanding of metabolic reprogramming during disease progression. Additionally, further validation of the remaining four 1-acyl-sn-glycero-3-phosphocholine metabolites is required to definitively identify the key regulators of cognitive function in HE progression. Finally, although we demonstrated the protective effect of GPC on cognitive function in HE rats, the precise molecular mechanisms underlying this neuroprotective effect remain unverified. We have proposed potential mechanisms based on existing literature; however, more systematic investigations—including targeted pathway inhibition, gene silencing—are warranted to elucidate the pathways through which GPC exerts its regulatory effects.

## Conclusion

During disease progression, patients with liver cirrhosis and HE exhibit alterations in multiple metabolite levels. Among these, GPC, PC(22:6), PC(22:5), PC(17:0), and PC(20:5) may play a cognitively protective role in HE progression. Exogenous GPC supplementation can partially improve spatial working memory and locomotor activity in HE rats, while also reducing hepatic granulocyte infiltration and BBB damage. However, the precise underlying mechanisms require further investigation.

## Data Availability

The raw data supporting the conclusions of this article will be made available by the authors, without undue reservation.
