# Supplementary material for: Serum metabolomics identifies GPC depletion in hepatic encephalopathy and its therapeutic potential for cognitive impairment
Source: Front Pharmacol. 2026 Apr 29;17:1807444. doi: 10.3389/fphar.2026.1807444 (PMC13167568; doi:10.3389/fphar.2026.1807444)
Supplement: Supplementary file 1 [file Supplementaryfile1.docx]

1. **Modeling Methodology:**

**HE Group Surgical Procedure:**

1. **Anesthesia Induction:** Rats were anesthetized with a standardized concentration of sevoflurane until reaching a surgical plane of anesthesia, followed by maintenance at a reduced concentration.
2. **Preoperative Preparation:** The abdominal area was shaved and disinfected, with strict aseptic technique (sterile drapes and gloves) to prevent infection.
3. **Surgical Approach:** A small midline upper abdominal incision was made, combining sharp and blunt dissection to minimize hemorrhage and tissue trauma.
4. **CBDL Method:**
   - The modified CBDL was identified by gentle visceral manipulation, exteriorized, and stabilized.
   - Double ligation was performed proximally and distally with surgical silk, followed by transection between ligatures.
   - Knot integrity was verified to prevent postoperative bile leakage.
5. **Closure & Recovery:** Tissues were repositioned and the incision closed. Postoperative monitoring included assessment of feeding, activity, and wound healing.

**Sham Group Procedure:**
Sham rats received identical anesthesia and prep procedures, but without CBD ligation. Instead:

- Visceral organs were gently probed with a sterile instrument for 5 minutes to simulate surgical stress.
- Organs were repositioned and the incision closed.
- Postoperative monitoring matched the HE group protocol.

**Control Group:**
Normal controls received no surgical interventions.

1. **Behavioral Testing Methods**

**Y-Maze Test**

The Y-maze consists of three arms arranged at 120° angles, with removable partitions at each arm entrance to restrict access.

**A. Spontaneous Alternation Test (Working Memory Assessment)**

**a. Primary Metric:** Spontaneous alternation rate (higher values indicate superior working memory)

**b. Calculation:** Spontaneous Alternation Rate (%) = (Number of Alternations)/ (Total Arm Entries – 2)×100. *(An alternation is defined as consecutive entries into all three arms without repetition)*

**c. Protocol:** Rats were acclimated to the testing environment for 2 hours to reduce neophobia. Each rat was placed at the distal end of one arm (facing away from the center) and allowed free exploration for 5 minutes.

**B. Novel Arm Test (Reference Memory Assessment)**

**a. Primary Metric:** Percentage of entries into the novel arm (higher values indicate better reference memory)

**b. Protocol:** Phase 1: One arm was blocked, and rats explored the remaining two arms for 5 minutes from a randomized start arm. Phase 2 (2 hours later): The blocked arm was opened, and rats re-explored all three arms from the same start arm for 5 minutes.

**Open Field Test**

The apparatus was a 100 × 100 × 40 cm arena with a central 70 × 70 cm square zone.

**a. Protocol:** Rats were acclimated for 2 hours before testing.

Each rat was placed in the center and allowed free exploration for 5 minutes.

**b. Metrics:**

Center Zone Duration: Longer stays indicate lower anxiety.

Total Distance Traveled: Reflects general locomotor activity and exploratory drive.

1. **Metabolite Screening Strategy:**

Based on our hypothesis, there exists one or a class of substances that progressively increase or decrease in healthy controls, cirrhosis patients, and HE patients, thereby contributing to or regulating cognitive changes in cirrhosis and HE patients. Therefore, we selected metabolites that were differentially expressed across all three comparison groups, totaling 16 metabolites. Since further validation is required, we prioritized metabolites with high identification confidence in metabolomics, specifically those classified as Level 1 and Level 2. This reduced the candidate metabolites for validation to 10, which are as follows: Glycochenodeoxycholic acid 3-glucuronide, PC(22:6(4Z,7Z,10Z,13Z,16Z,19Z)/0:0), PC(22:5(7Z,10Z,13Z,16Z,19Z)/0:0), PE(0:0/22:6(4Z,7Z,10Z,13Z,16Z,19Z)), PC(17:0/0:0), Glycocholic acid, PC(20:5(5Z,8Z,11Z,14Z,17Z)/0:0), PC(20:3(5Z,8Z,11Z)/18:2(9Z,12Z)), N,N'-Diallylpentobarbital, L-Valine.

KEGG-based metabolic pathway enrichment analysis demonstrated that the 10 differential metabolites were collectively enriched in 12 Level 3 metabolic pathways (*Supplementary Figure 2*), including Choline metabolism in cancer, Glycerophospholipid metabolism, Efferocytosis, Primary bile acid biosynthesis, Arachidonic acid metabolism, Linoleic acid metabolism, alpha- Linolenic acid metabolism, valine/leucine/isoleucine degradation, valine/leucine/isoleucine biosynthesis, Pantothenate and CoA biosynthesis, Aminoacyl-tRNA biosynthesis, and ABC transporters. Notably, three metabolites - Glycochenodeoxycholic acid 3-glucuronide, PE(0:0/22:6(4Z,7Z,10Z,13Z,16Z,19Z)), and N,N'-diallylpentobarbital - showed no enrichment in any metabolic pathways, which would hinder subsequent mechanistic investigations. Therefore, these three metabolites were excluded from further validation, leaving seven candidate metabolites for subsequent analysis.

According to the order of disease progression, namely Con→Cir→HE, the relative quantities of metabolites in each group were calculated. Meanwhile, using the fuzzy clustering principle of the R package Mfuzz, the characteristic metabolites were mapped to several clusters, and the membership value of each characteristic metabolite within its respective cluster was computed (the higher the membership value, the better it fits the current cluster). Among them, the changing trends of Cluster 3, Cluster 7, and Cluster 10 matched the anticipated variations of characteristic metabolites during disease progression (*Supplementary Figure 3*). Of the seven candidate metabolites initially proposed for validation, only glycocholic acid was not classified into these three clusters that aligned with our hypothesized disease progression and metabolite trend changes. Therefore, it was temporarily excluded from further validation, reducing the number of candidate metabolites to six.

Among the remaining six metabolites, L-valine was attributed to Cluster 7 and classified as a valine and its derivatives under organic acids and derivatives. The other five metabolites belonged to glycerophosphocholines within lipids and lipid-like molecules, showing close interconnections. These five glycerophosphocholine metabolites were all enriched in the two metabolic pathways with the smallest p-values—glycerophospholipid metabolism and choline metabolism in cancer. Therefore, the subsequent validation will focus only on these five glycerophosphocholine metabolites. Among these five metabolites, PC(20:3(5Z,8Z,11Z)/18:2(9Z,12Z)) was identified as a characteristic metabolite in Cluster 3, with its parent class being phosphatidylcholines. The remaining four differential metabolites (PC(22:6(4Z,7Z,10Z,13Z,16Z,19Z)/0:0), PC(22:5(7Z,10Z,13Z,16Z,19Z)/0:0), PC(17:0/0:0), and PC(20:5(5Z,8Z,11Z,14Z,17Z)/0:0), listed in descending order of membership values) were all attributed to Cluster 10, with their parent class being 1-acyl-sn-glycero-3-phosphocholines. PC(20:3(5Z,8Z,11Z)/18:2(9Z,12Z)) was upregulated in all three comparison groups. Its parent class, phosphatidylcholine (commonly known as lecithin), was also upregulated in all three groups within the choline metabolism in cancer and glycerophospholipid metabolism pathways. However, these two metabolic pathways were overall downregulated in the HE-vs-Cir and HE-vs-Con comparison groups, while showing mixed upregulation and downregulation in the Cir-vs-Con group. This suggests that the changes in phosphatidylcholine are not the primary driver of these pathway alterations. Given that phosphatidylcholine is a well-known major component of cell membranes, we hypothesize that, as the disease progresses, damage to hepatocytes and other cells leads to membrane disruption, resulting in the release of phosphatidylcholine and a gradual increase in its expression levels. Therefore, its changes may not be directly linked to cognitive function alterations. For this reason, we have tentatively decided not to include this metabolite in further validation.

Consequently, we are left with only four 1-acyl-sn-glycero-3-phosphocholine metabolites for further validation. From the pathway maps of choline metabolism in cancer and glycerophospholipid metabolism (*Supplementary Figure 2*), we observed changes in three closely related substances: phosphatidylcholine (PC, commonly known as lecithin), 1-acyl-sn-glycero-3-phosphocholine (lysophosphatidylcholine, LPC), and sn-glycero-3-phosphocholine (GPC). The four candidate metabolites all belong to (LPCs). Their downstream metabolic fate involves either conversion into PC via lysophosphatidylcholine acyltransferase (LPCAT) or hydrolysis into GPC by phospholipase A1 (PLA1). Since these four differential metabolites are partially metabolized into GPC, we plan to first validate the relationship between GPC and cognitive function during disease progression from cirrhosis to HE. Notably, the expression levels of these metabolites gradually decrease as the disease progresses, suggesting that they may play a protective role.

1. **Mini-Mental State Examination (MMSE)**

**a. Orientation (10 points)**

1. What is the **year**?
2. What is the **season**?
3. What is the **date**?
4. What day is it today?
5. What is the **day**?
6. What is the **month**?
7. What is the **city**?
8. What is the **street**?
9. What is the **building** (if applicable)?
10. What is the **floor** (if applicable)?

**b. Registration (3 points)**
Name three objects (e.g., apple, table, penny).
Ask the patient to repeat the names of the objects.
Score 1 point for each correct answer.

**c. Attention and Calculation (5 points)**
Ask the patient to **subtract 7** from 100, and then continue subtracting 7 for a total of 5 subtractions.
Alternatively, you can ask the patient to spell the word "WORLD" backwards.
Score based on the number of correct subtractions or correct letters in the word.

**d. Recall (3 points)**
Ask the patient to recall the three objects named earlier in the "Registration" section.
Score 1 point for each correct answer.

**e. Language (8 points)**

1. **Naming (2 points)**: Show two objects (e.g., pencil and watch) and ask the patient to name them.
2. **Repetition (1 point)**: Ask the patient to repeat the phrase: "No ifs, ands, or buts."
3. **Comprehension (2 points)**: Give the patient a simple written command, e.g., "Take a piece of paper in your right hand, fold it in half, and put it on the floor."
4. **Reading (1 point)**: Ask the patient to read and obey a written command, e.g., "Close your eyes."
5. **Writing (1 point)**: Ask the patient to write a sentence. The sentence should be meaningful.
6. **Drawing (1 point)**: Ask the patient to draw a figure (e.g., intersecting pentagons).

**Total Score: ____ / 30 points**

**Scoring Interpretation**

- 24 or above: Normal cognitive function.
- 18-23: Mild cognitive impairment.
- Below 18: Significant cognitive impairment.


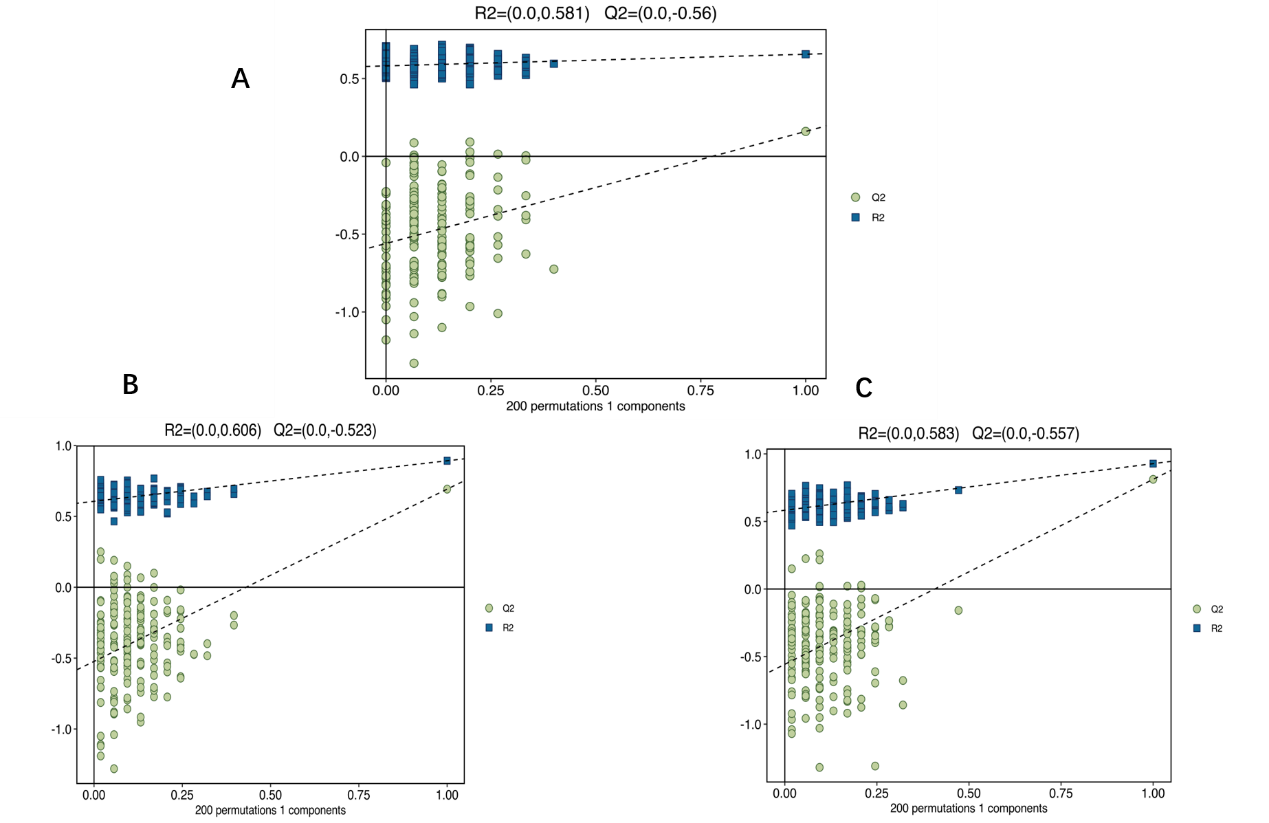


**Supplementary Fig. 1** Permutation plots for each comparison group. **(**A) Cir VS Con; (B) HE VS Cir; (C) HE VS Con. For all three comparison groups, the green Q² values on the left side were lower than the original points on the right, demonstrating the reliability of the corresponding OPLS-DA models without overfitting.


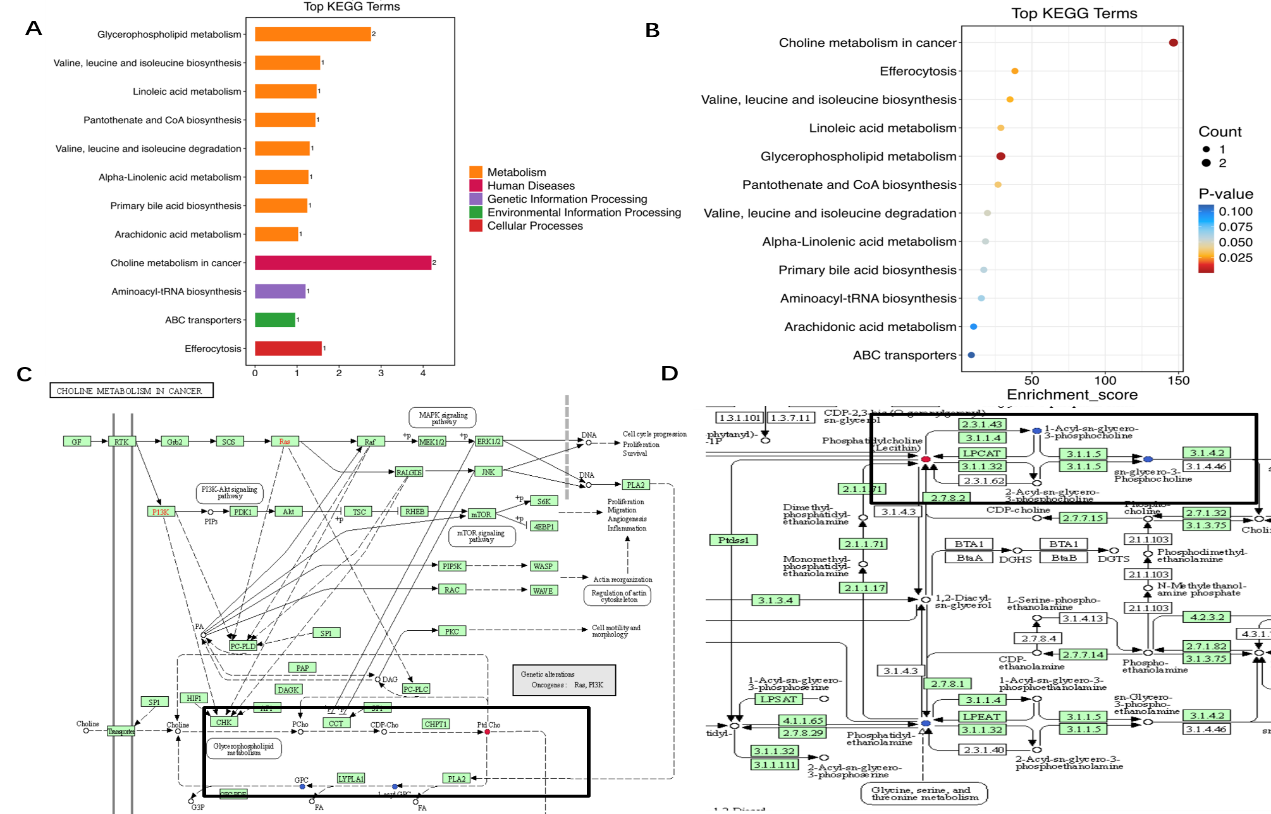


**Supplementary Fig. 2** KEGG pathway enrichment analysis graph. **(**A) KEGG Level 3 distribution plot of the 10 differential metabolites; (B) KEGG bubble plot of the 10 differential metabolites; (C) choline metabolism in cancer pathway; (D) glycerophospholipid metabolism pathway. In the pathway diagrams, red-highlighted metabolites represent significantly upregulated differential metabolites detected in the experiment, blue-highlighted metabolites indicate significantly downregulated differential metabolites, and the black box denotes the variation of GPC within the metabolic pathway.


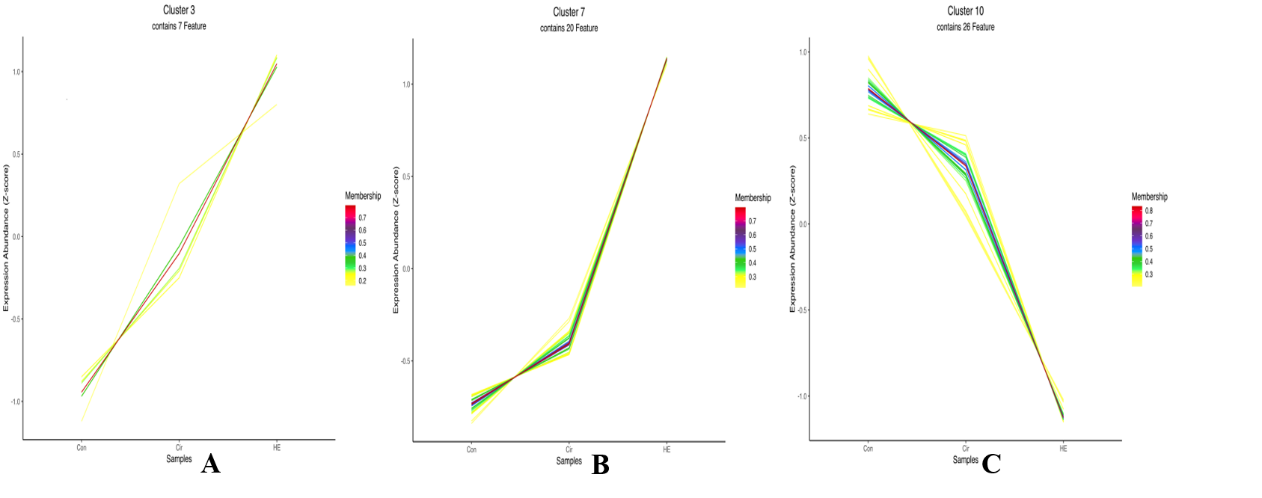


**Supplementary Fig. 3** Metabolite time-series trend analysis. Following the disease progression sequence (Con→Cir→HE), we calculated the relative abundance of metabolites across groups and performed fuzzy clustering analysis using the R package Mfuzz to categorize characteristic metabolites into distinct clusters. For each cluster, we computed membership values for the characteristic metabolites (with higher values indicating stronger association with the current cluster). Three clusters exhibited change patterns consistent with the anticipated trajectory of disease progression - specifically demonstrating gradual increase (A and B) or decrease (C) of specific metabolite classes along the disease continuum.


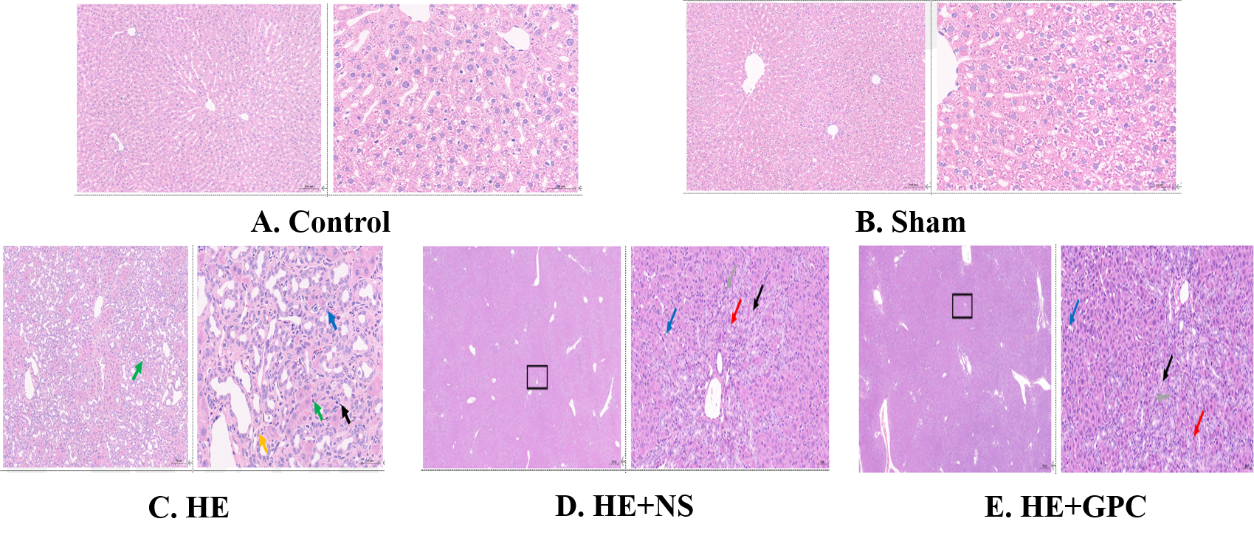


**Supplementary Fig. 4** HE results of the liver**.** Each group includes two micrographs: in Control, Sham, and HE groups, the left panel shows 100x magnification and the right panel shows 400x magnification, revealing indistinct hepatic lobule boundaries with increased neutrophils (green arrows), fibroblasts (yellow arrows), neoplastic epithelial cells (blue arrows), and necrotic debris (black arrows); in HE+NS and HE+GPC groups, the left panel displays 20x magnification and the right panel shows 200x magnification (black rectangle indicating zoomed area), demonstrating disrupted lobular architecture with prominent bile duct hyperplasia (black arrows), sparse fibroblast proliferation (gray arrows), occasional granulocyte infiltration (red arrows), and focal hepatocyte steatosis (blue arrows) containing cytoplasmic microvesicles.

**Supplementary Table 1** MS parameters

| Parameters | Cation | Anion |
| --- | --- | --- |
| Aux Gas Flow Rate (Arb) | 8 | 8 |
| Aux gas heater temperature (℃) | 350 | 350 |
| Capillary Temperature (℃) | 320 | 320 |
| Full ms resolution | 60000 | 60000 |
| Mass range (m/z) | 70-1050 | 70-1050 |
| MS/MS resolution | 15000 | 15000 |
| NCE/stepped NCE | 10,20,40 | 10,20,40 |
| Spray Voltage (V) | 3800 | -3000 |
| Sheath Gas Flow Rate (Arb) | 35 | 35 |
| S-lens RF level | 50 | 50 |
